# NURR1 Alterations in Perinatal Stress: A First Step towards Late-Onset Diseases? A Narrative Review

**DOI:** 10.3390/biomedicines8120584

**Published:** 2020-12-08

**Authors:** Laura Bordoni, Irene Petracci, Jean Calleja-Agius, Joan G. Lalor, Rosita Gabbianelli

**Affiliations:** 1 Unit of Molecular Biology and Nutrigenomics, School of Pharmacy, University of Camerino, 62032 Camerino, Italy; laura.bordoni@unicam.it; 2 School of Advanced Studies, University of Camerino, 62032 Camerino, Italy; irene.petracci@unicam.it; 3 Department of Anatomy, Faculty of Medicine and Surgery, University of Malta, MSD2080 Msida, Malta; jean.calleja-agius@um.edu.mt; 4 School of Nursing and Midwifery, Trinity College Dublin, 24 D’Olier Street, Dublin 2, Ireland; j.lalor@tcd.ie

**Keywords:** perinatal stress, NURR1, inflammation, late-onset diseases, early life

## Abstract

Perinatal life represents a delicate phase of development where stimuli of all sorts, coming to or from the mother, can influence the programming of the future baby’s health. These stimuli may have consequences that persist throughout adulthood. Nuclear receptor related 1 protein (NURR1), a transcription factor with a critical role in the development of the dopaminergic neurons in the midbrain, mediates the response to stressful environmental stimuli in the perinatal period. During pregnancy, low-grade inflammation triggered by maternal obesity, hyperinsulinemia or vaginal infections alters NURR1 expression in human gestational tissues. A similar scenario is triggered by exposure to neurotoxic compounds, which are associated with *NURR1* epigenetic deregulation in the offspring, with potential intergenerational effects. Since these alterations have been associated with an increased risk of developing late-onset diseases in children, NURR1, alone, or in combination with other molecular markers, has been proposed as a new prognostic tool and a potential therapeutic target for several pathological conditions. This narrative review describes perinatal stress associated with *NURR1* gene deregulation, which is proposed here as a mediator of late-onset consequences of early life events.

## 1. Introduction

Perinatal stress due to various environmental stimuli can have an impact on early fetal development, leading to long-term effects on cellular homeostasis [1,2,3,4]. Both prenatal and postnatal factors such as maternal nutrition, environmental pesticide exposure, stress, suboptimal antenatal care and neonatal trauma can cause epigenetic changes and impaired gene expression, especially at the neuronal level, with a consequent impact on fetal brain development and function [5].

According to the Barker hypothesis, in utero and postnatal stressors permanently program the structure and the physiology of the offspring, as a manifestation of the developmental plasticity to specific environmental stimuli [6]. This plasticity appears advantageous since it creates phenotypes that, once outside of the womb, are better matched to the environment that they are expected to enter into [6]. However, if in utero conditions do not match those following childbirth, this adaptive response could turn into a harmful mechanism. For instance, if the imprint left by a limited availability of nutrients during the prenatal stage is followed by overnutrition later in childhood, the risk of developing metabolic disorders increases, with consequent permanent changes in the metabolism of glucose-insulin established in the prenatal period [7]. The duration and timescale of exposure to various stimuli in early life are of key importance due to perinatal epigenetic plasticity. The interplay between environmental stimuli and genetic susceptibility in response to environmental stress is of a crucial importance in determining the final phenotype. Several genes play key roles in counterbalancing stress and maintaining cellular homeostasis. In the brain, the nuclear receptor related 1 protein (NURR1), a transcription factor able to modulate differentiation, survival and function of dopaminergic neurons, has been demonstrated to exert a neuroprotective role against neuropathological stress or insults. NURR1, as a glucocorticoid-responsive transcription factor, has an important endocrine regulatory role. It is a key factor in modulating the adaptive responses to stress by influencing the transcription of target genes in the hypothalamus-pituitary-adrenal axis (HPA), the major stress-responsive neuroendocrine system [8,9]. Changes in *NURR1* expression have been observed in neurodegenerative conditions such as Parkinson’s disease (PD) and Alzheimer’s disease (AD), as well as in stroke and in multiple sclerosis [10,11]. *NURR1* deregulation is also a causative factor for the onset of schizophrenia, through the modulation of genes associated with this pathology, particularly the dopamine D2 receptor co-expression gene set [12,13]. Considering epidemiological data on human cohorts [14,15,16] as well as the outcomes observed in PD animal models [17,18,19,20,21], it has been demonstrated that environmental stress during early life influences the programming of adult neuronal health. Thus, neonatal life represents the starting point during which the control of environmental stimuli can significantly drive the onset of neurodegeneration and other late-onset diseases.

This narrative review aims to describe the long-term effects of major environmental stressors occurring during the perinatal period and that affect *NURR1* gene regulation. PubMed database was used for the search of peer-reviewed original research articles in English, published up to October 2020, without including electronic early-release publications. Search terms included “Nurr1” or “Nr4a2”and “disease” or “early-life” or “perinatal or prenatal” or “stress” or “trauma” or “inflammation” or “epigenetics” or “environmental exposure”. The abstracts of retrieved citations were reviewed and prioritized by relevant content. Full articles were collected and secondary references from these articles were screened for inclusion.

This manuscript discusses how early determinants and maternal stress in perinatal life can modulate dopaminergic neuron homeostasis, as well as inflammatory and metabolic pathways, and affect health status later in life. The value of the early identification of risk factors lies in the fact that it may assist the introduction of prevention strategies aimed at reducing the burden of chronic diseases later in life. Consequently, it will assist policymakers to adopt appropriate clinical guidelines to prevent neuronal damage and inflammation-related diseases.

## 2. Early Determinants of Human Health

Increasing rates of prevalence of noncommunicable diseases (NCDs) (i.e., heart disease, diabetes, chronic respiratory diseases, cancer, PD, AD, mental illness, etc.) over recent decades, have been well-documented on a global scale. It is of particular concerning that the age of onset is reducing [22,23]. Major risk factors associated with the onset of NCDs are unhealthy diet, physical inactivity, alcohol consumption, smoking, air pollution and food xenobiotic exposure [24,25]. Considering the experimental, clinical and epidemiological evidence on the impact of early life on a wide range of NCDs, epigenetic processes occurring during the perinatal period have been identified as major mechanisms in the regulation of health later in life [26]. Human health can be programmed during prenatal and postnatal life as nutrition, life style, environment and genetics regulate gene expression and shape the phenotype. Starting from early prenatal life, parental exposure to healthy and unhealthy factors influence the child’s epigenome, driving long-term effects on the adult health [27]. Paternal body weight, maternal caloric overload, junk food consumption and malnutrition exert their impact not only on child body weight at birth, but also on his/her inflammatory responses. A correlation between prenatal maternal depression and cytokine levels has been observed in postmortem fetal brains in response to the maternal condition, suggesting that proinflammatory cytokine genes can be expressed in specific fetal brain regions and may influence their development [28,29,30].

Furthermore, maternal education can influence the duration of breastfeeding, which impacts the infant’s oral tolerance through immune modulation, the epithelial barrier function, the intestinal microbiome and body weight [31,32]. Shorter lengths of breastfeeding have been associated with increased proinflammatory responses, such as the production of interleukin 6 (IL-6) and C-reactive protein in the mother and in her offspring when reaching adulthood [33].

Social determinants of health, such as local neighborhood, social environment, exposure to chronic stress, education levels, socioeconomic status and access to health care, can cause epigenetic perturbations that influence disease susceptibility throughout life [34]. Social inequalities such as lower parental income/wealth, educational attainment, occupational social class, parental unemployment and lack of housing have been linked to unfavorable child health and development [35]. Poor housing conditions impact indoor air quality, leading, for example, to worsening asthma by epigenetic modulation [34]. Furthermore, in disadvantaged areas, the intake of healthy food is limited, with an excess of regular consumption of ultra-processed food, alcohol and tobacco, which contribute to the development of unhealthy phenotypes [34]. A low socioeconomical status, increased maternal weight and physical inactivity have been found to be related to children’s weight and height [36,37]. A direct correlation between family income and child health also exists. In the United States of America, children aged <17 years living in poor families are at an increased risk of suffering from poor health [38]. All in all, the social determinants of health have been defined by the World Health Organization as “the conditions in which people are born, grow, live, work and age” and “the fundamental drivers of these conditions” can affect health-related behaviors. Levels of family income and education are strongly associated with a wide range of health outcomes. Life expectancy in men and women and infant mortality rate are directly related to educational attainment. Parental education has an impact on children’s health because it influences dietary choices and exercise options early in life [38]. Finally, in addition to lifestyle-related determinants of health, traumatic events occurring in the perinatal life can also have a major impact on the child’s future health.

## 3. Stressful Events that Might Occur in Perinatal Life

Several stressful events might occur during the perinatal life, thus impacting on the health status of children both in early and later life, in particular as they are related to the development of the central nervous system (CNS). Prenatal exposure to inflammatory insults has been shown to lead to neurodevelopmental disorders [39]. In particular, maternal infection has been associated with long-term neurological and neuro-psychiatric morbidity in the offspring [40]. Maternal immune activation in animal models induces transgenerational effects on the brain and behavior [41]. Maternal chorioamnionitis is associated with cerebral palsy in the offspring, independently of other factors such as preterm delivery and birthweight [42]. Prematurity is associated with perinatal neuroinflammation and injury [43], and maternal inflammation has been identified as a major risk factor for premature birth. After birth, premature infants often require supplemental oxygen for survival, and this exposure can lead to additional inflammatory responses. Adults born preterm are at an increased risk of suffering from long-term conditions as a consequence of the severe disruption of the normal developmental maturation of organ systems. These adverse health problems, which tend to appear earlier in the pre-term-born population, include neurological and mental health problems, hypertension, diabetes, cardiac dysfunction and obstructive lung disease [44]. Instead, the risk of developing asthma and allergic diseases in adult life is higher in babies born by caesarean section [45,46]. Babies born by caesarean section have a reduced diversity of gut microflora when compared to babies delivered vaginally, and this seems to be the most likely explanation for the increase in allergic diseases, given the impact of gastrointestinal flora on the neonate’s immune system [47,48]. Maternal thyroid hormones are essential for normal neurodevelopment in the offspring, even after the onset of fetal thyroid function. This is particularly relevant for preterm infants who are deprived of maternal thyroid hormones following birth, who are at risk of suffering from hypothyroidism, and more likely to develop attention-deficit/hyperactivity disorder [49]. Rat models show that hypothyroid lactating females have a persistent low-quality milk, and both male and female hypothyroid offspring born of hypothyroid mothers gain less body mass with lower total adipose reserves and higher visceral reserves. The hypothyroid offspring also have higher levels of blood glucose, insulin and leptin, as well as dyslipidemia [50]. These long-term anthropometric effects have also been observed in humans [49]. Maternal diet has both short-term and long-term implications on fetal and child health. Maternal malnutrition can lead to micronutrient inadequacies and a suboptimal macronutrient balance [51,52,53]. For example, vitamin D deficiency together with maternal immune activation during development can induce schizophrenia-relevant dopaminergic abnormalities in the adult offspring of animal models [54]. Treating mothers with vitamin D could possibly lead to early neuroprotection to the fetus, since it has been shown to increase the number and the expression of mature Nurr1 mesencephalic dopaminergic neurons. Similar findings were observed in mothers with vitamin B deficiencies [55]. Bad eating habits can lead to maternal obesity with undesirable metabolism, which in turn influence the maternal health and, in the infant, lead to longer-term metabolic, neuropsychiatric and cognitive health consequences [56,57,58]. For instance, altered levels of plasma ceramides in the offspring of obese mothers have been implicated as early predictors of metabolic disease [59]. Maternal obesity has been implicated as being an independent risk factor of short- and long-term neuropsychiatric disorders in the offspring [60]. Low-grade inflammation is a central feature of pregnancies complicated by maternal obesity. This has also been observed in maternal type 2 diabetes, including gestational diabetes mellitus (GDM) [61]. Exposure in utero to maternal hyperglycemia, and consequent fetal hyperinsulinemia, carries not only several short-term consequences in the offspring, but also prompts metabolic imprinting that results in a greater risk of adverse long-term metabolic outcomes later in life. In particular, exposure in utero to maternal diabetes seems to influence long-term metabolic outcomes. The offspring of obese and/or mothers with diabetes carry a higher risk of obesity and type 2 diabetes, thus leading to a vicious cycle for future generations [62]. Exposure to toxins, including maternal smoking, is detrimental to the offspring. Animal models confirm that prenatal exposure to gestational nicotine before neurulation has a negative impact on the offspring’s neurodevelopment [63]. Epidemiological studies show that in utero exposure to maternal active and passive smoking has long-term neurological effects on the children [64,65]. This has also been demonstrated for other environmental toxins, such as perchlorate [66]. Similarly, the safety of anesthetic agents has been questioned due to the occurrence of apoptotic neurodegeneration and permanent cognitive deficiencies in immature animals after exposure to anesthetic agents [67].

## 4. NURR1: An Orphan Nuclear Receptor at the Interface between Neural Development, Inflammation and the Environment

NURR1, also called NR4A2, is a nuclear receptor and a transcription factor that belongs to the NR4A subfamily of nuclear receptors, which also includes NOR-1 and NUR77. NURR1 shares structural similarities with the other NR4A family members. It consists of a modulator domain at the N-terminus, referred to as the activation function (AF)-1, a central double zinc finger DNA-binding domain (DBD), a ligand-binding domain (LBD) composed of 12 α-helices and its transactivation-dependent AF-2 at the C-terminus [68]. Similar to the other two members of this subfamily, NOR-1 and NUR77, NURR1 falls within the category of orphan receptors, since no specific ligand has yet been identified [69]. Because of the steric bulk of several hydrophobic residues, NURR1 (as well as other NR4A family members) does not have a LBD cavity, which explains the difficulty in finding proper ligands that can directly activate NURR1 through its LBD [70]. Instead, *NURR1* transcriptional activity seems to rely on the AF-1 domain [71]. However, residues 592, 593, and 577 in the NURR1 LBD can be the site of interaction with some regulatory compounds [72,73]. For example, omega-3 docosahexaenoic acid has recently been shown to have high affinity for the NURR1 LBD, modulating *NURR1* transactivation [74]. NURR1 exists as an active transcription factor in both its monomeric and homodimeric forms. As a monomer, NURR1 binds the nerve growth factor-inducible-β-binding response element (NBRE; 5′-AAAGGTCA-3′), while as a homodimer, it binds the nur-response element (NurRE; 5′-TGACCTTT-n6-AAAGGTCA-3′), resulting in the activation of several genes, including the tyrosine hydroxylase (*TH*) and the dopamine active transporter (*DAT*) genes [75]. Indeed, *NURR1* is widely expressed in the CNS where it has a crucial role in the differentiation of midbrain dopaminergic (DA) neurons. *NURR1* is expressed during DA neuron differentiation in limbic areas and in the ventral midbrain where it regulates dopamine synthesis through proteins such as TH, DAT, vesicular monoamine transporter 2 (VMAT2) and RET receptor tyrosine kinase. Deficient expression of *NURR1* in developing mesencephalic dopaminergic cells impairs them with regard to expressing TH [76,77]. NURR1 deficiency in embryonic ventral midbrain cells impairs their migration and their ability to innervate striatal target areas [78]. Given its well-established role in the CNS, altered functionality of NURR1 has been also associated with neurodegeneration (PD in particular), but also with attention-deficit/hyperactivity disorder [79], schizophrenia and manic-depressive disorders [80]. Moreover, a de novo deletion-induced haploinsufficiency of NR4A2 receptors is implicated in neurodevelopmental alterations, in particular language impairment [81]. The role of NURR1 at the interface with environmental stimuli in the management of stressful events has been demonstrated [82,83]. There is evidence that NURR1 transcription factor plays a prominent role in adaptive responses to stress, regulating the transcription of target genes in the HPA axis [71]. Moreover, NURR1 activity seems to be enhanced upon the interaction with the glucocorticoid receptor (GR) [84]. Rapid increase in NURR1 mRNA expression has been measured in limbic and cortical brain structures related to coping with depression-like behavior in mice [85,86], suggesting that an increase in *NURR1* expression might be a compensatory mechanism to counteract the changes in forebrain dopamine transmission while coping with acute stress. The direct relationship of NURR1 with the environment is also suggested by its association with circadian rhythms and catecholamine production [87]. Prenatal stress modulates NURR1 inducing different outcomes along the life span of the male offspring, leading to changes in the reproductive system and spermatogenesis after puberty [8]. Beyond its well-known role in the development, function and maintenance of midbrain dopaminergic neurons [88], NURR1 can also be found in non-neuronal tissues such as synovial tissues, bone, endothelial cells, adrenal gland, hepatocytes and macrophages [83,89], where it mediates essential physiological processes, including adaptive and innate immune cell differentiation, metabolism and inflammation [90]. Thus, the nuclear receptor superfamily has been proposed as key transcription factors capable of modulating both immune and metabolic pathways. Since the discovery that NURR1 is not only involved in neurodegenerative disorders but also in inflammatory processes, growing attention has been directed to explore the potential role of NURR1 alterations in several inflammation-related diseases (including obesity and diabetes, atherosclerosis, cancer) [91,92,93,94,95]. In fact, the NURR1 receptor can be rapidly induced by a range of cytokines, suggesting that this receptor acts as a potential transcriptional mediator of inflammatory signals [83,96]. It has shown an anti-inflammatory function [97], but the exact molecular mechanisms have not been clearly elucidated yet. Recently, NURR1 has been shown to be responsive to nonsteroidal anti-inflammatory drugs [98]. The pleiotropic effects of NURR1 and its interaction with environmental factors have contributed to the proposal of this transcription factor as a mediator of late-onset consequences of early life events [83,99,100,101]. Next, we review the current knowledge on NURR1 alterations in early life, especially in association with the previously mentioned perinatal stressful events, and the potential implications in premature prevention of late-onset diseases.

## 5. Perinatal Stress Modulates *NURR1* Expression: From Early-Life Stress to Late-Onset Diseases

Differential regulations of *NURR1* expression have been demonstrated in association with several environmental exposures, especially in early life. In relation to metabolic health, it must be considered that in human fetal membranes and myometrium, as well as other cells and tissues, *NURR1* expression is rapidly and transiently induced by a wide range of stimuli, including hormones, cellular stress and inflammatory signals. Among these, obesity and GDM are of particular concern during pregnancy as they trigger low-grade systemic inflammation [102,103]. High levels of activated macrophages in the intestinal stroma of the placenta and circulating proinflammatory cytokines, such as TNF-α, IL-1β and IL-6, are observed in overweight women or women with GDM. In particular, TNF-α is considered a predictor of insulin resistance during pregnancy and has been correlated with fetal adiposity [104]. Interestingly, it was found that proinflammatory stimuli from IL-1β and TNF-α upregulate *NURR1* expression (as well as NUR77) in the placenta of women with GDM compared to body mass index-matched normal glucose tolerant pregnant women, even though the exact mechanism has not been elucidated yet [105,106].

Upregulated levels of cytokines are also observed in case of maternal depression [28]. Depressive disorders, anxiety and post-traumatic stress disorders are associated with significantly elevated levels of circulating proinflammatory cytokines, such as IL-6, TNF-α and IL-1RA [29,107,108,109]. This may be due to the activation of central and peripheral immune cells releasing cytokines, and to the activation of the stress response system of the HPA axis by proinflammatory cytokines [110]. The directionality of the related cytokine-depressive behavior is still under investigation. Inflammation, which is accompanied by cytokine signaling, may play a role in the pathophysiology of psychiatric disorders [111]. Nevertheless, changes in cytokines levels could also follow as a consequence of the psychiatric disorder, for example, being induced by treatments with psychopharmacological agents or by weight changes that accompany acute episodes of the disorder [112].

No association was found between pre-existing maternal obesity and placental *NURR1* expression. However, a positive correlation was found previously in adipose tissue, suggesting a tissue-dependent modulation of obesity-induced NR4A receptor expression [113]. Moreover, Veum and co-workers measured a strong upregulation of the NR4As in extreme obesity and normalization after fat loss, showing an altered adipose tissue expression of the NR4As in obesity [92]. Therefore, these stress-responsive nuclear receptors may modulate pathogenic potential in humans, and early-life trauma might stimulate their deregulation. In addition, human gestational tissues express NR4A receptors, which regulate the processes of parturition at term through the modulation of cytokines and growth factors [114,115,116]. *NURR1* (and *NUR77*) knockdown on primary human trophoblast cells resulted in decreased TNF-α induced IL-6 and IL-8 expression and secretion, revealing a possible proinflammatory effect of NURR1 in human placenta [105]. Inflammation has a central role during labor and delivery, because cytokines stimulate uterine activation via the NF-kB pathway inducing the release of prostaglandins [117]. *NURR1* (and *NUR77*) expression is upregulated in human fetal membranes and myometrium as a consequence of spontaneous labor at term, which can explain the expression of proinflammatory and prolabor genes associated with fetal membrane rupture and myometrial contractions [118]. However, a similar effect is driven by bacterial infections, which are responsible for most spontaneous preterm births (before 32 weeks gestational age) [119] due to the inflammatory response triggered by bacterial products in human gestational tissues. In fetal membranes, *NURR1* expression was upregulated by bacterial lipopolysaccharide, fibroblast-stimulating lipopeptide and peptidoglycan muramyl dipeptide, whereas flagellin also increased *NURR1* expression in the myometrium. The upstream mechanisms behind *NURR1* upregulation are not clear yet; however, NF-kB activation seems to be involved [118]. By disrupting the normal developmental maturation of organ systems, preterm birth may result in long lasting adverse effects in adult age. Increased blood pressure, reduced insulin sensitivity, impaired vascular growth, chronic kidney disease (especially in the case of intrauterine growth restriction or neonatal acute kidney injury) and significant chronic airway obstruction are the most common adverse consequences connected to preterm birth that persist through adulthood [43]. Concerning CNS health, prenatal or early postnatal stress are considered risk factors for the development of psychiatric disorders, addiction and the ability to cope with stress. Prenatal stress strongly impacts fetal brain development in rats. Rats exposed to different types of stress during the last week of pregnancy give birth to offspring with anomalies in neuronal development and brain morphology which persist through adulthood [120,121]. The underlying mechanism has been thought to be most likely due to changes in D2-type dopamine (DA) neurotransmission induced by prenatal stress [122]. *NURR1* expression in dopaminergic neurons starts at embryonic day 10.5 before the appearance of the dopaminergic marker enzyme, TH (at embryonic day 11.5) and continues during adulthood [123]. A homeostatic function has been attributed to NURR1 in the case of stress. Levels of NURR1 were found to be increased in the ventral tegmental area of prenatally stressed adult offspring, most likely as a compensatory mechanism to counteract the reduction in dopamine levels observed as a consequence of prenatal stress [8,124]. A similar NURR1 increase was observed in cortical brain regions and the limbic system, including cornu ammonis-3 (CA3) of the hippocampus in mice, as a compensatory response to acute stress [85]. Montes et al. has shown that even if the hippocampus may be vulnerable to stress, it may also have enough plasticity to cope with stress. To test the resilience to stress of the hippocampus, NURR1 was downregulated in prenatally stressed (PS) and nonprenatally stressed (NPS) male rats, through the bilateral administration of NURR1 anti-sense oligodeoxynucleotide (ODN) into their hippocampal CA3 region. Then rats were exposed to an acute stressor (forced swimming test, FST) to analyze their behavioral responses. After the ODN treatment, NPS rats showed a depressive-like behavior manifested through immobility, while PS rats showed active behaviors (resilience). These findings suggest that prenatal stress might induce brain modifications that promote resilience to acute stress in adulthood [125]. Given the central role of NURR1 in the development of dopaminergic neurons, prenatal exposure to neurotoxic compounds, such as pesticides, could be implicated in its deregulation leading to the onset, later in life, of neurological disorders, such as PD. Exposure to atrazine (ATR), a volatile and water-soluble compound used as a herbicide worldwide, during pregnancy and lactation has been associated with decreased expression of NURR1 in offspring, together with changes in the expression of VMAT2, which controls the transport and reuptake of DA. The consequent decrease in DA levels in the striatum confirm that early-life exposure to ATR alters the dopaminergic system by modulating *NURR1* expression [126]. Additionally, early-life exposure to permethrin (PERM), a pyrethroid compound largely used for outdoor/indoor pest control and as anti-woodworm agent, induces dopaminergic neuronal disorders in adult life, through the alteration of Nurr1 expression levels [100,101,127]. Of note, early-life exposure to PERM seems to have intergenerational effects, most likely due to epigenetic mechanisms [128]. An increased DNA methylation at the promoter region of the dopamine-specifying factor, Nurr1, has been observed in the sperm of first-generation offspring of these mothers. In the ventral midbrain of second-generation offspring, the effect is further associated with reduced mRNA levels of Nurr1 [41]. The effects in the later life of early NURR1 perturbation are endorsed by a body of evidence. Remarkably, it has been demonstrated that maternal smoking and early postnatal exposure to nicotine alter children’s behaviors and increase their propensity for drug abuse later in life, by altering the dopamine-mediated reward system [129]. This most likely occurs due to the nicotine-mediated circuit activation during development. In fact, studies on mice show that neonatal exposure to nicotine primes midbrain neurons to express NURR1; subsequent nicotine re-exposures in adulthood induce primed neurons to acquire the dopaminergic phenotype responsible for nicotine-mediated neurotransmitter plasticity [129]. In addition, prenatal exposure to infections is a known risk factor for the development of neuropsychiatric disorders, especially schizophrenia and autism [130,131]. However, it seems that other risk factors, in particular genetic factors, should be concomitant to developing severe neuronal disorders. Brain and behavioral consequences of prenatal infection-induced immune activation are exacerbated (synergistic effects) in offspring with genetic predisposition to dopaminergic abnormalities, in particular NURR1 deficiency [132]. NURR1 polymorphisms may also be implicated in the etiology of disorders characterized by altered dopaminergic signaling, such as attention-deficit/hyperactivity disorder, schizophrenia and PD. Thus, NURR1 may represent a future candidate gene to study the genetic predispositions to several neuropsychiatric disorders [79,130]. Furthermore, preclinical studies on rodents and nonhuman primates have questioned the safety of anesthetic agents used to relieve pain in the process of childbirth or surgical procedures. It emerged that under common clinical conditions, these chemical agents have a neurotoxic effect on the developing brain and can also induce long-term neurobehavioral abnormalities [133]. In particular, the use of sevoflurane in pregnant women seems to strongly impact fetal brain development. Sevoflurane impairs hippocampal CNS proliferation and differentiation through the upregulation of miR-183 and the downregulation of NURR1. The result is the progressive degeneration of the fetal brain, with long-term deficits in hippocampal-dependent learning and memory [133]. Finally, several studies also support the hypothesis of the intermediary role of inflammation and perinatal trauma underpinning the link between early-life exposure, NURR1 alterations, and CNS impairment later in life. Indeed, NURR1 expression may play a significant role in the modulation of brain development, especially in the case of a combination of maternal inflammation and premature birth. Premature infants often rely on supplemental oxygen for survival, which may represent an additional source of inflammation leading to neurodevelopmental impairment. Lallier et al. [134] found decreased numbers of oligodendrocytes and increased numbers of microglia in mice exposed to both maternal inflammation and neonatal hyperoxia. They hypothesized that alteration of NURR1 expression in the perinatal period could be responsible for the detrimental effects of the two combined sources of inflammation, bacterial infections and hypoxia [134]. Table 1 summarizes the main targets and modulators of NURR1.

## 6. NURR1: An Early Biomarker and Novel Target for Prevention of Chronic Diseases?

Given the expression of NURR1 not only in the CNS but also in other tissues, such as adipose tissue, liver, skeletal muscles and heart tissues, a perturbation of its functionality can have a broad spectrum of consequences for human health, from neurological and psychiatric disorders to metabolic diseases. NURR1 is mainly known for its primary role in the development of dopaminergic neurons; however, it has been shown to be a significant dual actor in the inflammation process. While there is evidence of the anti-inflammatory behavior of NURR1 [135], other findings have associated NURR1 expression levels with increased proinflammatory cytokines, in particular in pathologies such as PD and type 2 diabetes, suggesting a potential of NURR1 in the etiology of these conditions [11,93]. Even though further research to clarify the mechanistic effects of NURR1 is needed, the usage of NURR1 expression as a biomarker has been proposed, at least for those conditions in which NURR1 deregulation has been established. The assessment of NURR1 levels in blood gave good results in aiding in the diagnosis of PD and monitoring disease progression when measured in association with mir-132 or cytokines [136,137]. Other findings also suggest NURR1 as a biomarker for early diagnosis or diseases’ progression [93,138]. Additional studies investigating the role of NURR1 in predicting long-term effects of perinatal stress are warranted in order to extend the usage of NURR1 as a biomarker for other relevant clinical conditions.

In addition, NURR1 has been suggested as a potential pharmacological target for diseases characterized by its deregulation [139,140]. As an example, it has been demonstrated that the GR can act as a transcriptional regulator of NURR1, suggesting that glucocorticoids might be used to regulate NURR1 expression [87]. At present, a significant number of research projects aimed at identifying new NURR1 ligands for drug development are underway [141,142,143].

Moreover, knowledge that NURR1 expression can be regulated by modifiable factors (i.e., nutritional status) might pave the way for potential applications of nutrigenomics [144] in the early prevention of the previously mentioned conditions through strategies aimed to improve nutrition in the perinatal period and in early childhood.

## 7. Conclusions

The interactions between NURR1 and environmental factors, especially during early fetal development, are well-documented and are implicated in a variety of late-onset consequences for human health [99]. When considering that an intergenerational transmission of NURR1-mediated effects has been hypothesized [41,128], the ability of external stressors to control of NURR1 expression gains even more importance. This is of particular significance in the context of the prenatal and early neonatal periods. Further investigations to explore the role of NURR1 either alone or in combination with other molecular markers as a noninvasive biomarker, aiding in the prevention, diagnosis and evaluation of disease severity or response to treatments for several pathological conditions, should be considered.

## Figures and Tables

**Table 1:** Main targets and modulators of Nuclear receptor related 1 protein (NURR1).

NURR1 Targets (T) and Modulators (M)	References
Hypothalamus-pituitary-adrenal axis (T)	[8,9,71]
Dopamine D2 receptor, tyrosine hydroxylase, dopamine active transporter, glucocorticoid receptor (T)	[12,13,75,76,77,84,85,122,123,124,125,126]
Environmental stress during early life (M)	[8,14,15,16,17,18,19,20,21,82,83,99,100,101,129,130,131,132,133,134]
Circadian rhythm and catecholamine production (T)	[87]
Micronutrient intake (M)	[55,56,57,58]
Omega-3 docosahexaenoic acid (M)	[74]
Adaptive and innate immune cell differentiation, metabolism and inflammation in various cells (T)	[83,90,91,92,93,94,95,96,97,98,105,135]
IL-1β and TNF-α (M)	[29,104,105,106,107,108,109,110]
Obesity (M)	[92,113]
Human gestational tissues (T)	[114,115,116,118]
Permethrin (M)	[41,100,101,127,128]
